# The impact of feedback during formative testing on study behaviour and performance of (bio)medical students: a randomised controlled study

**DOI:** 10.1186/s12909-019-1534-x

**Published:** 2019-04-03

**Authors:** D. H. J. Thijssen, M. T. E. Hopman, M. T. van Wijngaarden, J. G. J. Hoenderop, R. J. M. Bindels, T. M. H. Eijsvogels

**Affiliations:** 1 Radboud Institute for Molecular Life Sciences, Radboud University Medical Center, Nijmegen, The Netherlands; 2 Department of Physiology (392), Radboud Institute for Health Sciences, Radboud University Medical Center, PO box 9101, 6500 HB Nijmegen, The Netherlands; 3 Research Institute for Sport and Exercise Sciences, Liverpool John Moores University, Liverpool, UK

**Keywords:** Feedback, Formative testing, E-learning, Medical education, Blended learning

## Abstract

**Background:**

A potential concern of formative testing using web-based applications (“apps”) is provision of limited feedback. Adopting a randomised controlled trial in 463 first year (bio) medical students, we explored if providing immediate, detailed feedback during “app”-based formative testing can further improve study behaviour and study performance of (bio)medical students.

**Methods:**

Students had access to a formative testing “app”, which involved 7 formative test modules throughout the 4-week course. In a randomised order, subjects received the “app” with (*n* = 231, intervention) or without (*n* = 232, control) detailed feedback during the formative test modules.

**Results:**

No differences in app-use was found between groups (*P* = 0.15), whereas the intervention group more frequently reviewed information compared to controls (*P* = 0.007). Exam scores differed between non−/moderate−/intensive- users of the “app” (*P* < 0.001). No differences in exam scores were found between intervention (6.6 ± 1.1) versus control (6.6 ± 1.1, *P* = 0.18). Time spent studying was significantly higher compared to previous courses in moderate- and intensive-users (*P* = 0.006 and < 0.001, respectively), but not in non-users (*P* = 0.55). Time spent studying did not differ between groups (*P* > 0.05).

**Conclusions:**

Providing detailed feedback did not further enhance the effect of a web-based application of formative testing on study behaviour or study performance in (bio)medical students, possibly because of a ceiling-effect.

## Highlights

- This study examined whether adding feedback to formative testing using smartphone-based applications (“apps”) can increase knowledge retention.
- We found that smartphone-based applications improve study performance and increase time spent studying in moderate- and intensive-users of the “app”.
- Providing detailed feedback to “app”-users was not associated with further improvement of study behaviour or study performance, possibly because of a ceiling-effect of the “app”.

## Background

Previous studies have demonstrated that knowledge retention can be successfully achieved by performing repeated sessions of studying [1, 2]. Formative testing, which refers to the method of test-enhanced learning, adopts the use of frequent tests to improve retention of information. This strategy successfully improves the processes of learning and retention of knowledge through multiple ways [3]. When matched for an equal amount of time, formative testing is more effective in knowledge retention compared to re-studying material [4, 5]. Another explanation for the success of formative testing relates to the larger spread of students’ study activities, which allows them to identify (and subsequently specifically focus on) areas of weakness [3].

The frequently reported underuse of formative testing opportunities [6, 7] may be effectively reduced by the introduction and integration of e-learning in higher education. Indeed, e-based learning tools are both effective and well-appreciated across various settings of (bio)medical education [8–11], ultimately enhancing study behaviour and study performance [12]. One potential concern of internet-based formative testing relates to providing detailed feedback. In addition to simply providing correct answers [13–15], information on why a certain answer is (in)correct may improve study performance [16]. Little work explored whether providing detailed feedback during formative testing can further enhance study results.

Therefore, in this study we build on a recently validated internet-based app for formative testing in first year students (bio)medicine [12], and explored if providing detailed feedback during formative testing can further improve study performance of biomedical students. We hypothesized that students who receive detailed feedback in response to incorrect answers will perform better during the final exam. Since our previous study demonstrated that the app improves study behaviour [12], our secondary aim was to examine whether providing detailed feedback during formative testing would further stimulate study behaviour.

## Methods

### Population

We included 324 medicine students and 139 biomedical sciences students who registered for the course “Circulation and Respiration”. This 4-week course is part of Year 1 for both medicine and biomedical sciences of the Radboud university medical center. Before the course, all students received information about the study and the app, although students were not informed that some will receive the feedback-option of the app. Prior to participation of our study we received (written) informed consent from all our participants. The educational advisory board of the Radboud university medical center provided approval of the proposed study. We adhered to the international guidelines from the Declaration of Helsinki regarding our study design and the collection and analysis of data.

### Experimental design

Following a block-randomisation, based on the “working group” (~ 15 students who follow all teaching-related activities together), students were randomly assigned to using the “Physiomics to the next level”-app with (“intervention”) or without (“control”) receiving feedback. When questions were answered incorrectly, feedback on the background of the specific question was provided. The exam of the 4-week “Circulation and Respiration”-course consisted of a written examination which included multiple-choice questions only. The final exam grades were scored from 1 (i.e. lowest score) to 10 (i.e. highest score), whilst students passed this exam when they obtained a score of ≥5.5. After the exam, all students were requested to fill in a questionnaire on their study behaviour and the use of the app.

### App design

**Fig. 1:**
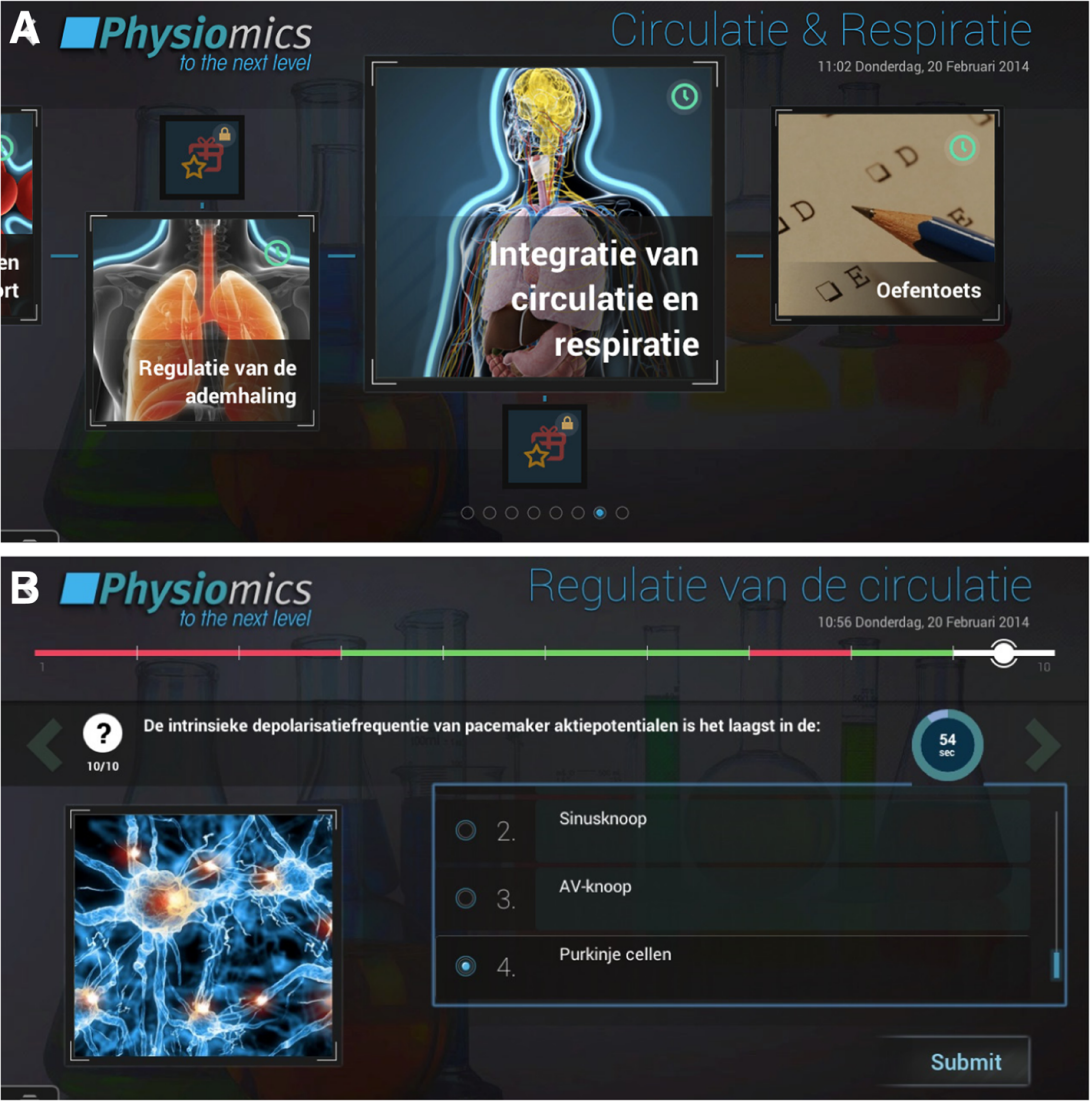
Screenshots of the app adopted by the students. **a** represents an overview of some of the topics that were covered in the 4-week course. **b** is one of the 10 questions related to a specific topic

Recruitment for participation in the study was performed first prior to the start of the course through sending an email to all students, but also by providing information on the study on the virtual learning environment (i.e., Blackboard). After the start of the course, recruitment was performed by providing information on the study during the first lecture and first interactive lecture of the course. Upon agreement for participation, an email was sent with a personal password to allow access to the app. The app was designed as an open-source HTML-based application (https://eduweb.science.ru.nl). In designing the app, we ensured that access was possible for major operating systems and devices (e.g. cell phones, tablets, desktops and laptops) [12] (www2.physiomics.eu/app). A general impression is provided in Fig. 1. In short, the app provided access to a tutorial course (for familiarization), and to 7 course-specific modules (10 multiple-choice questions + mock examination). The questions of each module could only be answered for 72-h (excluding weekend days) to stimulate students to evenly spread study-load. When students correctly answered 7 out of 10 multiple-choice questions, 5 additional questions were unlocked. Questions remained available for review purposes at later time points.

Feedback regarding the answers to the questions in the app was provided directly by means of a green checkmark (for a correct answer) or a red cross (for an incorrect answer). No further feedback was provided in case of a correct answer (i.e., green checkmark). In case a wrong answer was given, the app-users (i.e., control) received a pop-up with information on relevant pages in their course-guide and textbook where they could search for background information that is relevant to this question. The students within the intervention group received immediate, detailed additional information on the right answer and information on why the other answers were incorrect.

### Data-collection

To provide detailed insight into the use of the app, several parameters were logged during the 4-week intervention (e.g. time spent and answer to each question, number of questions answered correctly). In addition, since students were allowed to review back previous questions and answers, we also logged the time students spent on reviewing back the answers. Finally, we recorded the examination grades.

Immediately after the exam, participants were instructed to fill in questionnaires regarding their study behaviour and the app-intervention. Regarding self-assessed study behaviour, students estimated the time (in hours) spent studying per week across the 4-week course. This information on study behaviour was provided for the intervention-course (i.e. Circulation & Respiration), but also for the preceding 4 courses. Importantly, the set-up and duration of these 4 preceding courses was comparable. These data were used to assess study behaviour during the 4-week course, but also to compare study behaviour during the present course that adopted the app versus preceding courses that did not adopt any type of (e-learning based) formal testing.

### Statistical methods

Statistical analyses were performed using the Statistical Package for the Social Sciences (IBM SPSS Statistics for Mac, Version 23.0. Armonk, NY: IBM Corp.). To examine the “use” of the app, a student was classified as a “non-user” (0 modules completed), “moderate user” (1–4 modules completed) or “intensive user” (5–7 modules completed). We presented all quantitative data as means ± standard deviation (SD), whilst categorical variables were presented as a percentage. Differences in study performance (i.e. dependent variable) between the intervention versus control groups (“feedback”) were compared between the non-, moderate- and intensive users (“user”) using a two-way repeated measures analysis of variance. We adopted Greenhouse-Geisser corrections for multiple comparisons. In addition, we compared study behaviour (i.e. dependent variable) between both groups (intervention versus control: “feedback”) across the 4 week course (“weeks”) using a two-way repeated measures analysis of variance. A three-way repeated measures analysis of variance was used to examine if study behaviour across the 4-week course from “Circulation and Respiration” differed compared to previous courses (“course”). Adopting similar statistical procedures, we tested potential differences in exam grade between user groups. Finally, binary logistic regression analysis was adopted to examine the effect of the app on the risk to fail for the final exam. Odds Ratios are presented with 95% confidence intervals (CI). Results were considered significant when *P* < 0.05.

## Results

### Participants

A total of 476 students were eligible to use the app. Students were equally distributed across the control group (*n* = 238) and intervention group (n = 238). Students that did not take the final exam were excluded from the data analysis (*n* = 13), leading to a final study population of 232 and 231 students for the control and intervention group, respectively. The majority of the students was female (65% female, 35% male) and studied medicine (70% medicine, 30% biomedical sciences) (Table 1). No differences between the control and intervention groups were found in sex (66% female vs 63% female, *P* = 0.44) or the ratio of students medicine vs biomedical science (71% vs 69%, *P* = 0.62). A total of 71% of the students used the app.

**Table 1:**
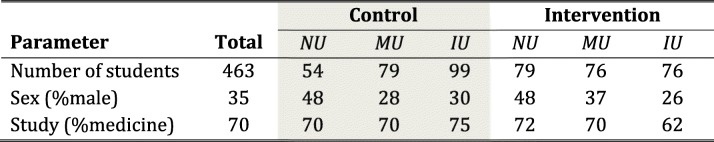
Group characteristics of the study cohorts. The cohorts of students using the “app” without feedback (Control, *n* = 232, grey) and with feedback (Intervention, *n* = 231) were divided into non-users (NU), moderate users (MU) and intensive users (IU)

### Study behaviour

The hours spent on studying gradually increased over the 4-week course (Table 2). For non-users, moderate-users and intensive-users, we found that the increase in hours spent studying was not different between the control versus intervention group (Table 2). When compared to previous courses, study hours were not different during the current course for the non-users (Table 3). In contrast, moderate and intensive users of the app spent more time studying during the current course compared to previous courses that did not use formative testing (Table 3). Adding detailed feedback to the app did not significantly alter study behaviour (Table 3).

**Table 2:**
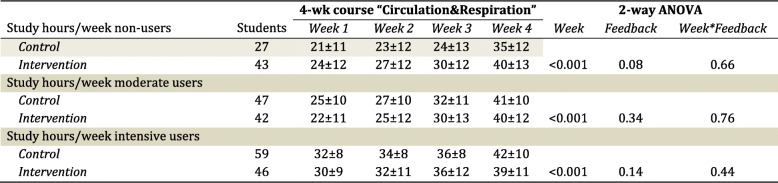
Self-assessed study behaviour (presented as the hours per week spent studying) across the 4-week Circulation & Respiration-course for students using the app (control, *n* = 133, grey) or app+feedback (intervention, *n* = 131). Study behaviour was presented for students after being divided into non-users (NU), moderate users (MU) and intensive users (IU) of the app. A 2-way ANOVA was performed to examine whether the time spent studying across the 4-week course differed between controls versus intervention

**Table 3:** Study behaviour (presented as the hours per week spent studying) across previous courses (average of 4 preceding courses) and the Circulation & Respiration-course for students using the app (control, *n* = 125) or app+feedback (intervention, *n* = 131). Study behaviour was presented for students after being divided into non-users (NU), moderate users (MU) and intensive users (IU) of the app. A 3-way ANOVA was performed to examine whether the time spent studying differed between the Circulation&Respiration-course versus previous courses (‘Course’), but also if adding feedback altered study behaviour across the 4-weeks (‘Course*Feedback*Time-interaction)

Study hrs/wk. non-users	Students	4-wk course				3-way ANOVA
		Week 1	Week 2	Week 3	Week 4	
Control (previous courses)	27	22 ± 11	23 ± 11	24 ± 13	35 ± 11	Course: P = 0.55
Control (Circ-Resp)	27	21 ± 11	23 ± 12	24 ± 13	35 ± 12	Feedback: *P* = 0.09
Intervention (previous courses)	42	24 ± 9	26 ± 10	28 ± 10	39 ± 11	Course*Feedback: *P* = 0.42
Intervention (Circ-Resp)	42	24 ± 12	28 ± 12	30 ± 11	40 ± 13	Course*Feedback*Time: *P* = 0.70
Study hrs/wk. moderate users						
Control (previous courses)	47	25 ± 9	27 ± 10	31 ± 9	38 ± 10	Course: P = 0.006
Control (Circ-Resp)	47	25 ± 10	27 ± 10	32 ± 11	41 ± 10	Feedback: *P* = 0.34
Intervention (previous courses)	40	22 ± 11	25 ± 11	28 ± 12	37 ± 12	Course*Feedback: *P* = 0.65
Intervention (Circ-Resp)	40	22 ± 11	25 ± 12	30 ± 13	40 ± 12	Course*Feedback*Time: *P* = 0.92
Study hrs/wk. intensive users						
Control (previous courses)	57	31 ± 9	33 ± 9	35 ± 9	39 ± 11	Course: P < 0.001
Control (Circ-Resp)	57	32 ± 8	34 ± 8	36 ± 8	42 ± 10	Feedback: *P* = 0.04
Intervention (previous courses)	43	26 ± 9	28 ± 10	30 ± 10	36 ± 11	Course*Feedback: *P* = 0.01
Intervention (Circ-Resp)	43	29 ± 9	31 ± 11	35 ± 12	39 ± 11	Course*Feedback*Time: *P* = 0.13

### Study performance

**Fig. 2:**
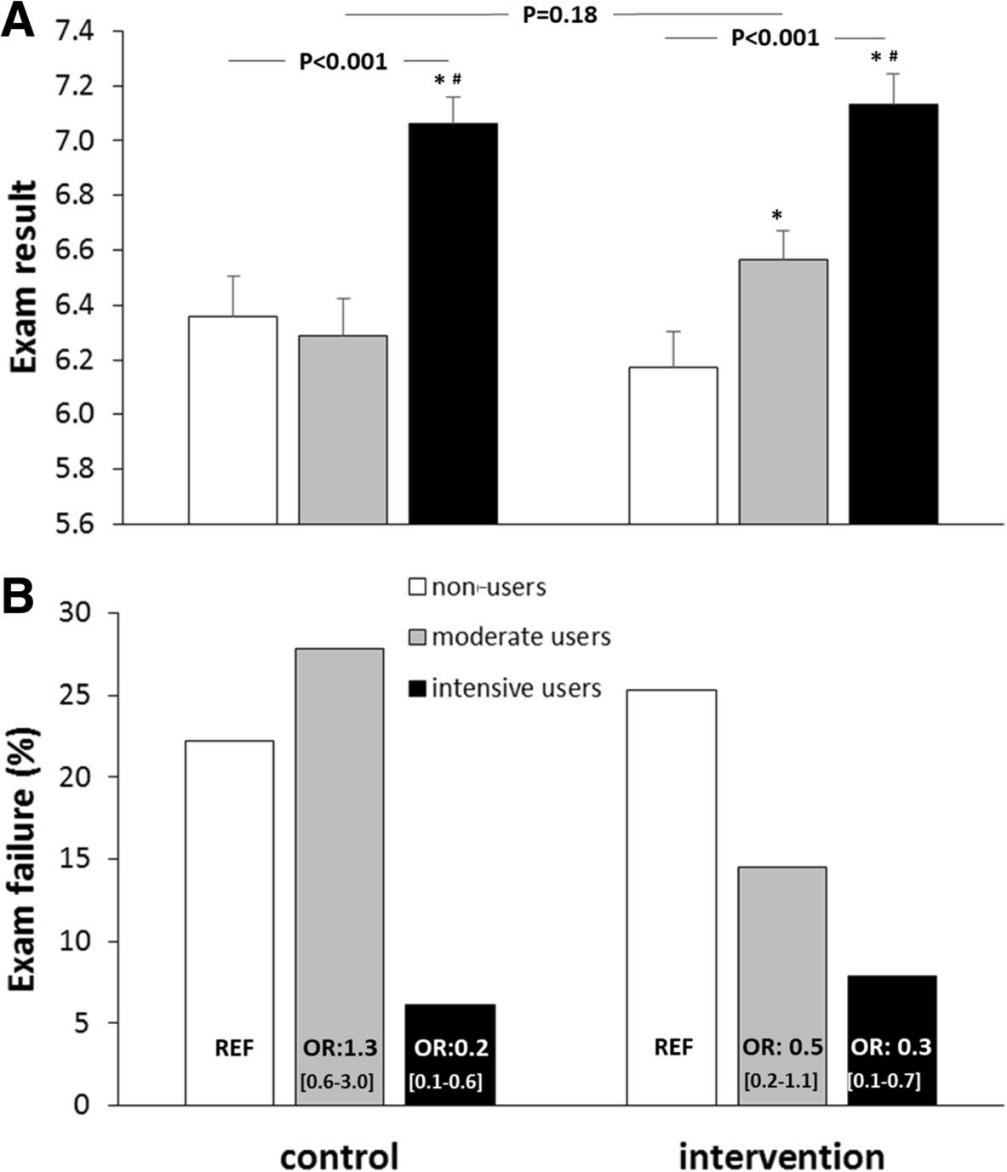
**a** Grades on the final exam subdivided by non-users (white bars), moderate users (grey bars) and intensive users (black bars) for the app-users (Control) and app + feedback-users (Intervention)**.** Data are presented as means ± SEM. *P*-value refers to a one-way ANOVA. * *p* < 0.05 compared to non-users, ^#^ p < 0.05 compared to moderate users. **b** Relative number of students who failed their exam subdivided by non-users (white bars), moderate users (grey bars) and intensive users (black bars) for the app-users (Control) and app + feedback-users (Intervention)

Students achieved a mean score of 6.6 ± 1.1 on the final examination of the course. Intensive users in the control group (*P* < 0.001), but not moderate users (*P* = 0.72), scored significantly better compared to non-users during the final exam (Fig. 2a). In the intervention group (*n* = 231), both moderate (*P* < 0.05) and intensive users (P < 0.001) scored significantly higher on the final exam compared to non-users and compared to each other (*P* = 0.001) (Fig. 2a). When using a 2-way ANOVA, no significant differences were observed in exam results between app- versus app + feedback-users (‘intervention’: *P* = 0.59), whilst this effect was similarly present between non-, moderate- and intensive- users (intervention*use: *P* = 0.18, Fig. 2a).

In total, 16.6% of all students failed to pass their final exam, which was not different between the intervention (16.0%) and control group (17.2%, P = 0.72). Nevertheless, we found that non-users more frequently failed their exam (24.1%) compared to moderate users (21.3%; OR 0.85, CI 0.49–1.48) and intensive users (6.9%; OR 0.23, CI 0.11–0.47). Trends were similar for the control and intervention groups (Fig. 2b).

### Practical use and evaluation

The average use of the app across the 4-week course was not different between the control and intervention groups (27 min:16 s ± 15 min:6 s versus 24 min 52 s ± 14 min 59 s, *P* = 0.15). However, the intervention group more frequently reviewed information (10 min:31 s ± 10 min:6 s) compared to the control group (7 min 45 s ± 8 min 3 s, *P* = 0.007). The respondents to the questionnaire (*n* = 264) indicated that they felt that the app improved their study behaviour (44.6%) and positively affected their exam preparations (63.6%), whilst 74.8% would like the app to be implemented in future courses. Importantly, the grading of these aspects did not differ between study arms (*P* = 0.98, 0.73 and 0.78, respectively).

## Discussion

The present study reinforces the ability of an internet-based app, providing a series of formative tests across a 4-week course, to improve exam results and study behaviour compared to non-users in first year (bio)medical students. More importantly, adopting a randomised controlled trial, we explored the impact of adding detailed feedback to the formative tests. In contrast to our hypothesis, providing feedback during formative testing did not alter study performance or study behaviour. Although these data confirm the benefits of internet-based formative testing in (bio)medical students, a ceiling effect may explain why adding feedback did not further improve study performance and study behaviour when using app-based formative testing.

The internet-based app for formative testing was used by 71% of the students, which is in line with previous work adopting similar technology for formative testing in biomedical teaching [10, 12, 17–19]. A majority of the students provided positive feedback on the app, in that 3 out of 4 students would like the app to be implemented in future courses. The potency of adding feedback to the app is supported by the observation that the intervention group more frequently reviewed information compared to the control group. This further supports and highlights the importance of providing feedback in learning environments. More importantly, the moderate- and intensive-users of the app spent more hours studying per week compared to previous courses. Since this difference was absent in non-users, the larger time spent studying by the app-users is most likely caused by the internet-based formative testing. In addition, the app-users also reported better study results, with intensive app-users demonstrating a 13.3% higher score than the non-users. The magnitude of improvement in the intensive app-users is in line with previous studies (e.g. 8–14%) when adopting formative testing under (un)controlled conditions [4, 12, 20, 21]. Despite these marked benefits of formative testing, the intensive app-users showed significant baseline differences in the time spent studying. This observation is in agreement with previous work [22, 23], in that (intensive) app-users users are higher achievers than the non-users. Although this baseline difference must be considered, our data confirm the value of adding internet-based formative testing to a blended learning situation in students (bio)medicine to improve study behaviour and study results.

After initial implementation of our formative testing-app in the 4-week course Circulation & Respiration [12], students frequently commented on the request for detailed feedback on the incorrect answers. Supported by previous work on the potential added value of providing detailed feedback [16], the app was updated by providing detailed information in case of incorrect answers. However, in contrast to our hypothesis, feedback did not further improve effects on study behaviour, study performance and/or appreciation of the app. Although Wojcikowski and Kirk found a positive impact of detailed feedback, their study was importantly limited by their design since interventions were compared between subsequent academic cycles (year 1: app, year 2 app+feedback). Subsequent years can lead to different scores, independent of the intervention used. Indeed, we found a 4% higher mean exam score compared to previous year, i.e. when we introduced the “app” (i.e., 6.37). A difference in exam scores during subsequent academic cycles, therefore, should be interpreted with caution.

One potential explanation for our findings is that the app already provided sufficient feedback, i.e. the correct answer and reference to the correct answer in case of an incorrect answer. Simply providing the correct answer has been demonstrated to significantly enhance study performance [13–15]. In addition to providing the students with an incorrect answer with (more) detailed information, no other changes were made in the app and/or course. Possibly, a ceiling effect may not have been achieved yet in the moderate users. Our study was not properly powered and designed to test this specific hypothesis, but future studies seem warranted to explore whether feedback could enhance the learning effects in the moderate-users. Another explanation for our results is that students had regular opportunities for verbal feedback (e.g. interactive lectures), including verbal feedback on app-based questions. This is relevant, as a previous study indicated that verbal feedback was associated with better study outcomes compared to written feedback [24]. A final consideration regarding our observation is that the type of feedback may affect its efficacy on learning, although previous work found that the type of feedback is more important during verbal rather than written feedback [25, 26]. At least, our results suggest that, when already providing minimal feedback (i.e., correct answers + references to where in the text books to find the answers), additional detailed feedback did not significantly affect study behaviour and exam results in first year students (bio)medicine, potentially due to a ceiling effect.

Although we adopted a strong design (randomized controlled trial) in a large cohort (*n* = 463), some limitations must be considered. Students were not informed about the aim of this study. During the evaluation, some app-users indicated that they used the app+feedback version from their friends and/or used the app+feedback together with others. Since we have no information on the exact number of students who followed such approach, we cannot exclude that this affected our main conclusions. Another potential weakness was the presence of baseline difference in study behaviour between non-, moderate- and intensive-user groups. Students who used the app showed better study behaviour, but also performed better based on historical examination grades compared to the non-users [12]. However, these baseline differences likely similarly affected the results in the control and intervention groups. Finally, from the app-users, one may hypothesize that adding feedback to formative testing may be more beneficial in low-to-moderate achievers rather than the high-achievers. For this reason, we repeated our analysis and added study behaviour (e.g. hours spent studying during previous courses) as a co-variate, but this did not alter the main outcomes of the analysis (data not shown). This suggests that adding feedback to the app did not impact study performance, independent of study behaviour in previous courses.

## Conclusions

In conclusion, our study further substantiated that 1st year students (bio)medical sciences who use electronic-based application of formative testing spend more hours studying and obtained higher grades on the final examination compared to non-users. More importantly, providing immediate, detailed feedback to students on the questions answered incorrectly (rather than providing information on where to find the correct answer), did not further improve exam performance, study behaviour and/or appreciation. Possibly, a ceiling effect of adding formative testing within a blended-learning environment in (bio)medical teaching may explain why providing additional information did not further enhance study performance and/or study behaviour.

## Abbreviation

App: Application
